# Inter-evaluator heterogeneity of clinical diagnosis for locally advanced esophageal squamous cell carcinoma

**DOI:** 10.1007/s10388-017-0580-x

**Published:** 2017-05-23

**Authors:** Yasuo Hamamoto, Masanori Nojima, Yu Aoki, Takeshi Suzuki, Kenta Kawasaki, Kenro Hirata, Yasutaka Sukawa, Akira Kasuga, Hirofumi Kawakubo, Hiroya Takeuchi, Koji Murakami, Hiromasa Takaishi, Takanori Kanai, Yuko Kitagawa

**Affiliations:** 1 Keio Cancer Center, Keio University School of Medicine, 35 Shinanomachi, Shinjukuku, Tokyo, Japan; 2 Center for Translational Research, The Institute of Medical Science Hospital, The University of Tokyo, Tokyo, Japan; 3 Division of Gastroenterology and Hepatology, Department of Internal Medicine, Keio University School of Medicine, 35 Shinanomachi, Tokyo, Japan; 4 Division of Surgery, Keio University School of Medicine, 35 Shinanomachi, Shinjukuku, Tokyo, Japan; 5 Department of Radiology, Keio University School of Medicine, 35 Shinanomachi, Shinjukuku, Tokyo, Japan

**Keywords:** Heterogeneity, Esophageal cancer, Resectability

## Abstract

**Background:**

Identifying clinical resectability of locally advanced esophageal squamous cell carcinoma (ESCC) is important, although inter-evaluator heterogeneity (IEH) could exist, especially in borderline resectable (BLR) cases. To investigate the extent of heterogeneity, we conducted clinical diagnostic imaging questionnaires.

**Materials and methods:**

Five cases with clinical T3 or T4 cases, which were treated with neo-adjuvant triplet chemotherapy followed by surgery, were selected as the model. These cases were divided into two groups: curative resected cases (#1–#3) and non-curative resected cases (#4 and #5). Only imaging slides were shown without any information about patient characteristics or clinical course. The evaluators consisted of surgeons (staff and non-staff), medical oncologists, and an imaging radiologist; a total of 25 medical staff answered the questionnaire. Two questions (1: clinical T stage before chemotherapy, 2: resectability after chemotherapy) were answered. Occupational differences were assessed by comparing the results to the imaging radiologist.

**Results:**

IEH was observed for clinical diagnosis before chemotherapy in one case (clinical T4: 52%, clinical T3: 48%). In the other cases, most evaluators diagnosed them as clinical T4, with 76–88% agreement. IEH for clinical resectability after chemotherapy was relatively small. Occupational IEH was observed in both before and after chemotherapy.

**Conclusion:**

IEH in decisions about treating BLR cases in ESCC should be considered in clinical practice. Multi-disciplinary teams are essential to overcome this problem.

## Introduction

The prognosis of locally advanced esophageal squamous cell carcinoma (ESCC) is generally poor, especially when the cancer is unresectable [1]. Definitive chemoradiotherapy with more than 60 Gy irradiation is the standard of care for advanced cases [2–4]. Several other treatment options, including resection, exist in general Japanese practice [5], although surgical removal is controversial [6, 7]. Neo-adjuvant chemoradiotherapy with 30–50 Gy with selected surgery if possible is classically preferred in some institutions [8–10], since the clinical decision as to whether it is resectable is difficult. Some unresectable cases convert resectable after completion of neo-adjuvant treatment; however, this strategy is not beneficial for non-curative cases. Patients with these finally unresectable cases also fail to complete the definitive radiation dose.

Recently, triplet chemotherapy with docetaxel, cisplatin (CDDP), and 5-fluorouracil (DCF) has been reported to produce a remarkable response for T4 disease [11–13]. The initial selection of intensive chemotherapy is beneficial when selecting the right patients for surgery. This strategy still has a curative option (salvage definitive chemoradiotherapy) if it fails to expedite curative surgery. However, this strategy also has several weaknesses. The most important problem with this strategy is consideration of borderline resectable (BLR) cases. As far as we know, there are no previous reports about a clear definition of BLR in ESCC.

Differential diagnoses for T stage are mainly judged using computed tomography (CT). The classical criteria for clinical T4 are considered the gold standard for advanced cases [14], although clinical diagnoses of marginal cases depend on the evaluator. Institutional heterogeneity may exist in these diagnoses, especially in BLR cases. Inter-evaluator heterogeneity (IEH) needs to be examined in more institutions with multiple specialized targets. The Japan Esophageal Oncology Group (JEOG) compared inter-institutional heterogeneity for locally advanced esophageal cancer. It failed to show any survival differences among the selected institutions [15]. However, precise analyses of IEH in real-world setting for locally advanced ESCC were not conducted. To clarify IEH, we conducted a clinical diagnostic imaging questionnaire study on medical staff (surgeons, medical oncologists, and an imaging radiologist) of BLR cases of ESCC.

## Patients and methods

### Study procedure

**Fig. 1:**
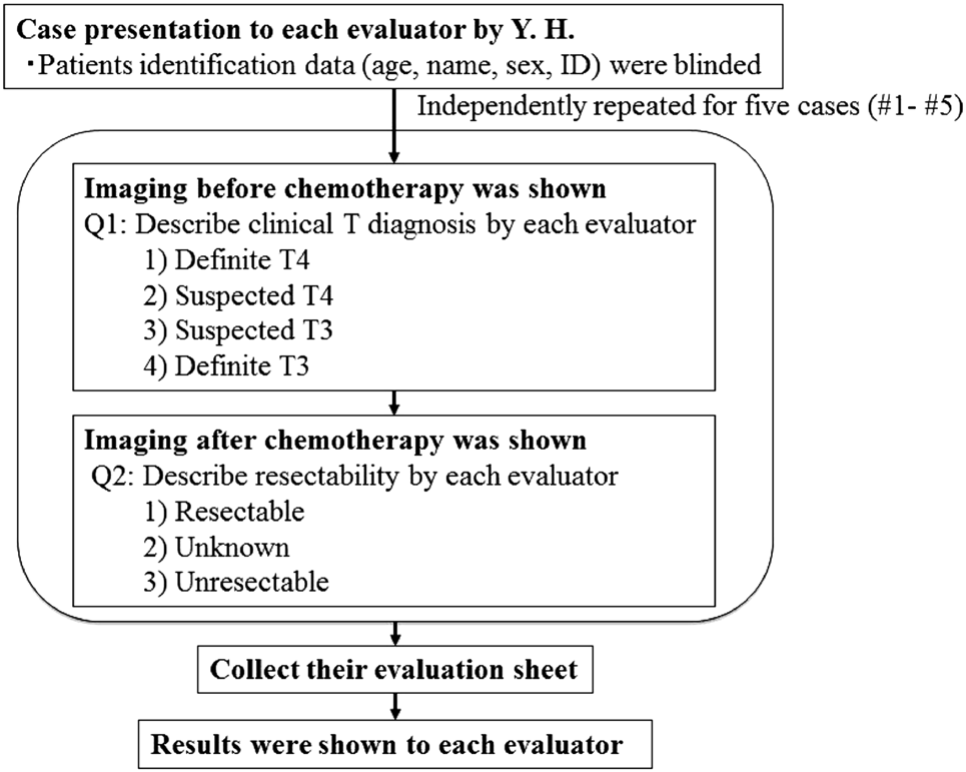
Protocol schema of the study

**Fig. 2:**
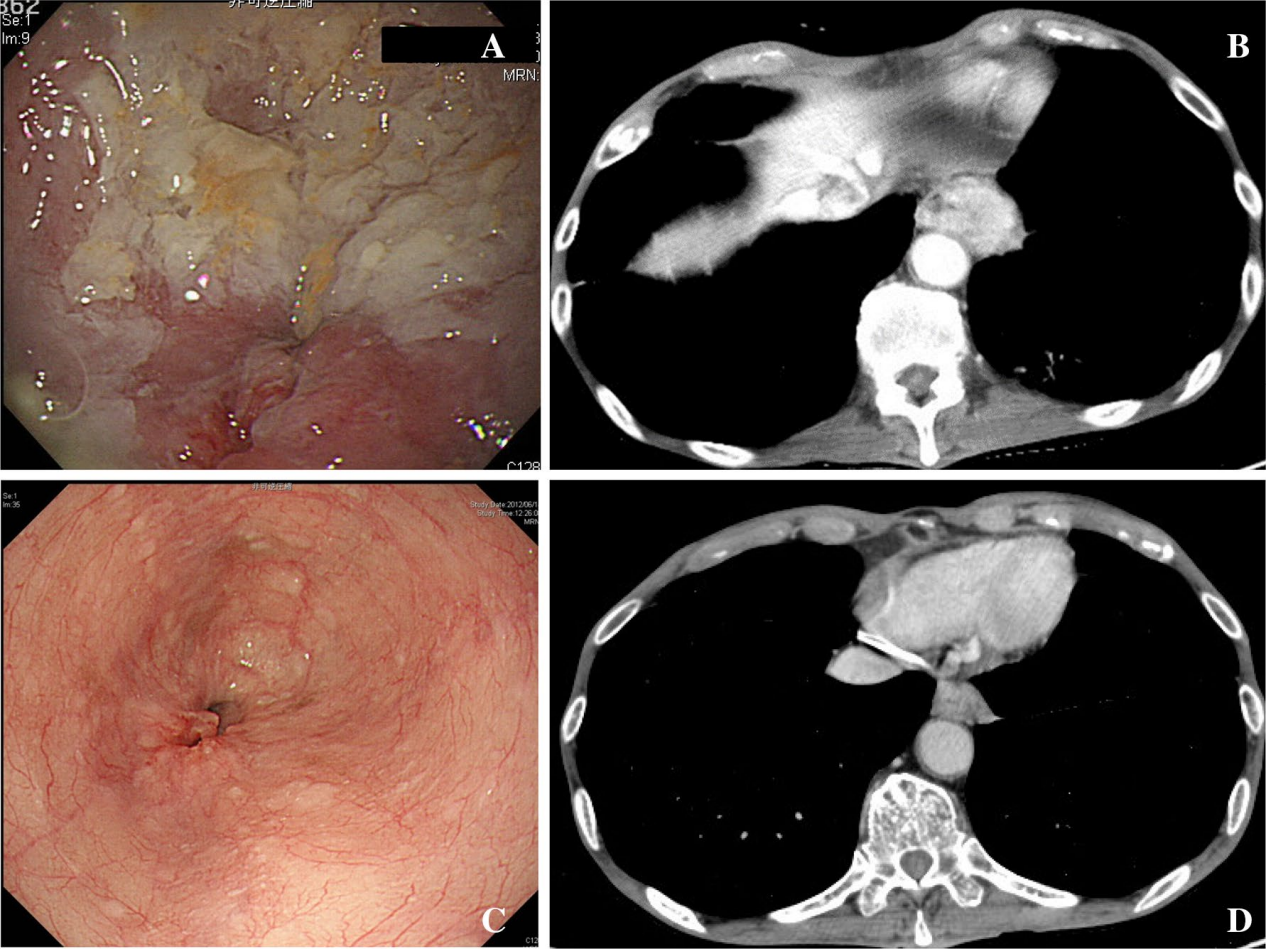
Case #1. This case selected as clinical T3 case. Adhesion to aorta or not is important differential diagnosis, although it was not as wide as T4. This case was finally resectable after chemotherapy. **a** Endoscopic findings before chemotherapy. **b** Computer tomography before chemotherapy. **c** Endoscopic findings after chemotherapy. **d** Endoscopic findings before chemotherapy

**Fig. 3:**
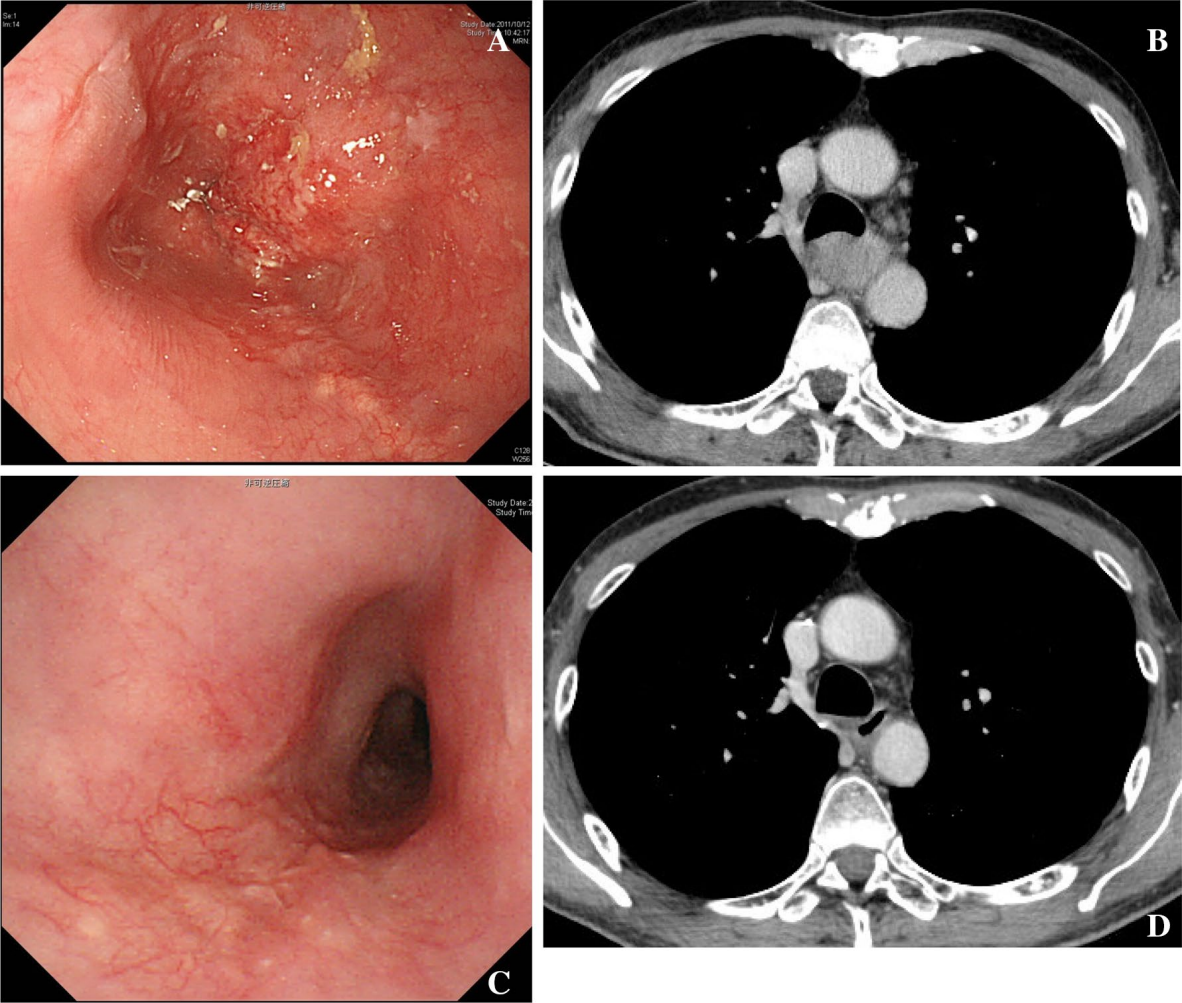
Case #2. This case selected as typical BLR case (relatively T4). Deformity of bronchus was evident. This case was finally resectable after chemotherapy. **a** Endoscopic findings before chemotherapy. **b** Computer tomography before chemotherapy. **c** Endoscopic findings after chemotherapy. **d** Endoscopic findings before chemotherapy

**Fig. 4:**
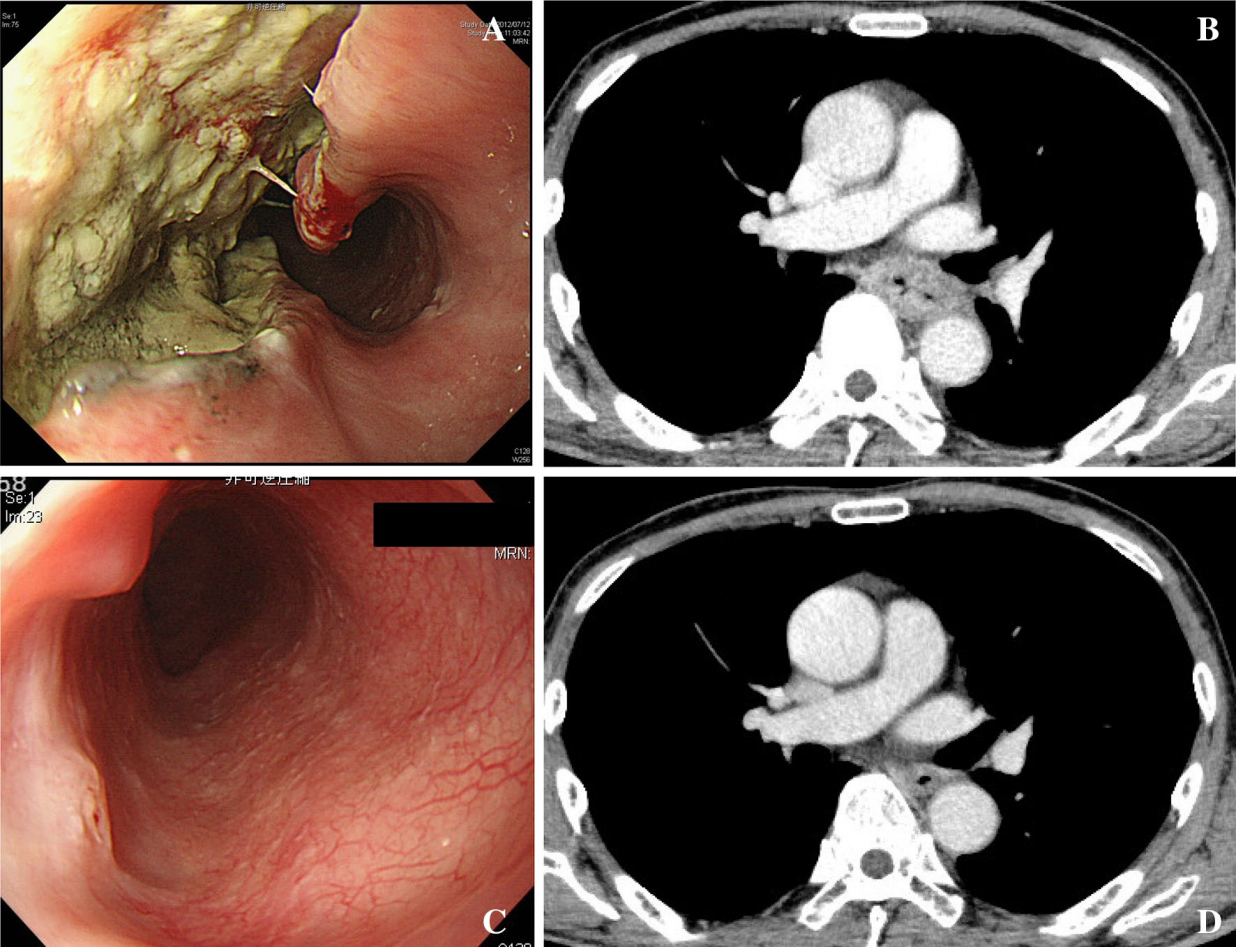
Case #3. This case selected as typical BLR case (relatively T4). Deformity of bronchus was evident. This case was finally resectable after chemotherapy. **a** Endoscopic findings before chemotherapy. **b** Computer tomography before chemotherapy. **c** Endoscopic findings after chemotherapy. **d** Endoscopic findings before chemotherapy

**Fig. 5:**
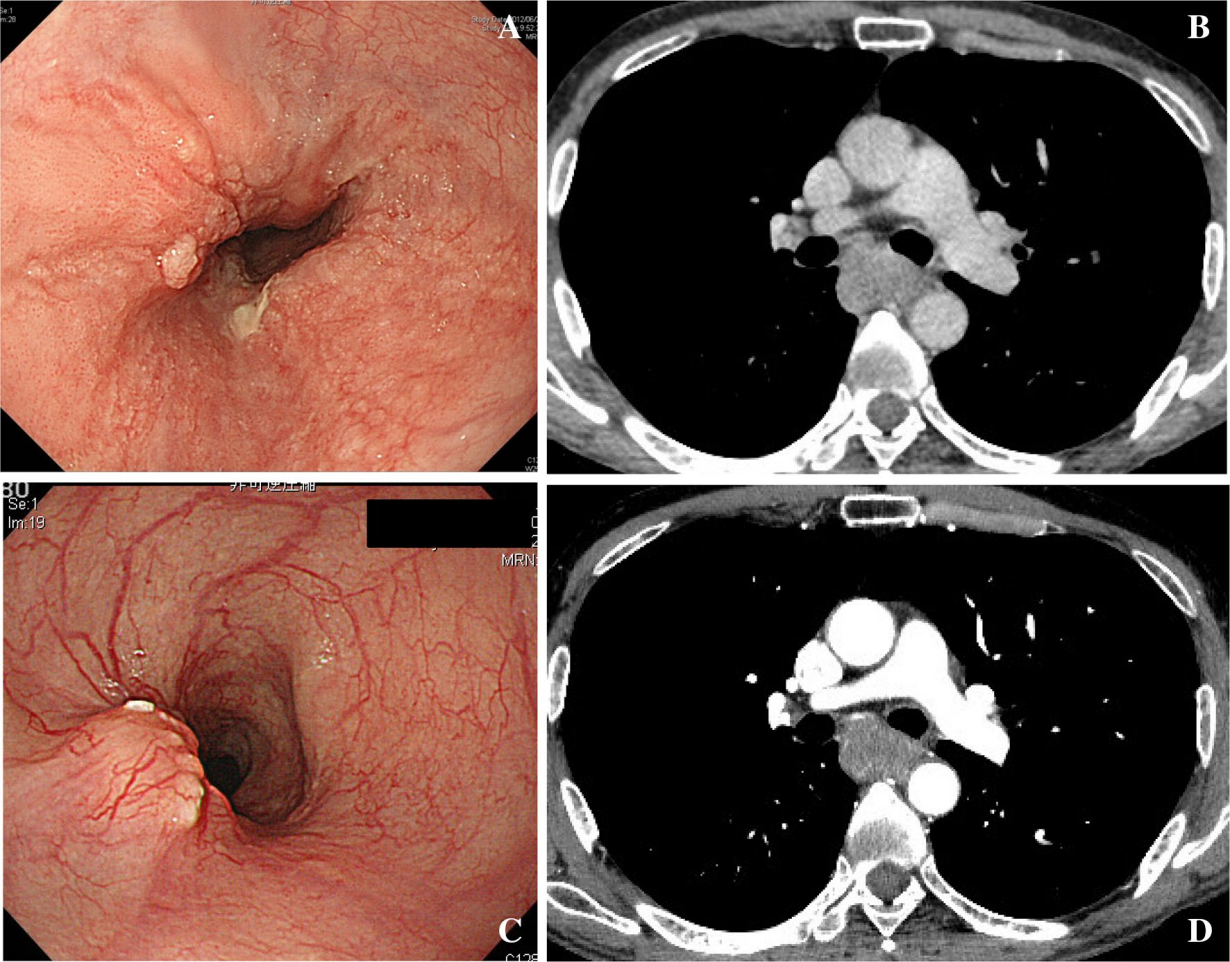
Case #4. This case selected as typical BLR case. This case fails to remove completely after chemotherapy. **a** Endoscopic findings before chemotherapy. **b** Computer tomography before chemotherapy. **c** Endoscopic findings after chemotherapy. **d** Endoscopic findings before chemotherapy

**Fig. 6:**
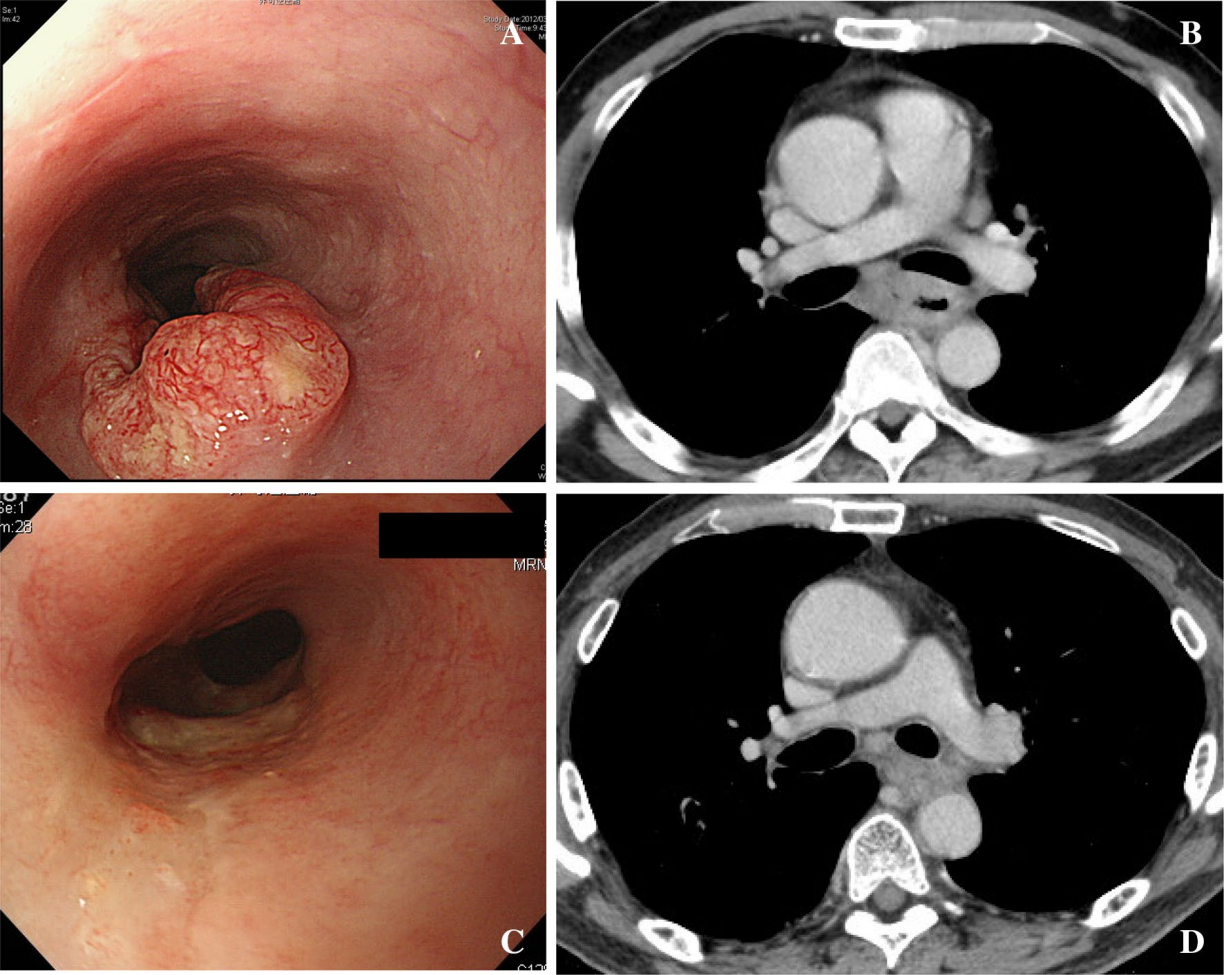
Case #5. This case selected as BLR case. Although adhesion area was limited, this case fails to resect completely after chemotherapy. **a** Endoscopic findings before chemotherapy. **b** Computer tomography before chemotherapy. **c** Endoscopic findings after chemotherapy. **d** Endoscopic findings before chemotherapy

The overall study procedure is shown in Fig. 1. Five cases with clinical T3 or T4 (Table 1; Figs. 2, 3, 4, 5, 6) were selected by the primary investigator (Y. H.), who did not answer the questionnaire. The reasons for selection were as follows: case #1 (Fig. 2): typical T3 case and initially judged as resectable; cases #2 (Fig. 3) and #3 (Fig. 4): typical BLR cases that were finally converted to resectable; and cases #4 (Fig. 5) and #5 (Fig. 6): typical BLR cases that were finally unresectable cases. All cases were treated with triplet chemotherapy, including DCF. They were evaluated after completion of 2 or 3 cycles of chemotherapy as to whether they were resectable. One case (#4) received subsequent chemoradiotherapy. All cases underwent attempted esophagectomy, although two cases (#4 and #5) could not achieve curative surgery and the final diagnoses were pathological T4b (Bronchus or Aorta). The other three cases (#1, #2, and #3) were finally diagnosed as resectable.

**Table 1:** Cases of presentation: blinded information to evaluators

Case	c-stage (TNM ver.7)	Location (thoracic)	Pre-operative treatments	Curative resection	P-stage	Pathological grade	Comments
#1	c-stage IIA cT3N0M0	Lower	DCF 3 cycles	Yes	pT3N1M0	Grade 1a	T3 case, aorta invasion need to discuss Adhesion area is not as wide as T4 Initially judged as resectable
#2	c-stage III cT4b(Br)N1M0	Middle	DCF 3 cycles	Yes	pT3N1M0	Grade 1a	Typical BLR case (relatively T4) Deformity of bronchus were evident Finally curative resection
#3	c-stage IVa cT4b(Br)N4M0	Middle	DCF 3 cycles	Yes	pT3N0M0	Grade 1b	Typical BLR case (relatively T4) Deformity of bronchus were evident Finally curative resection
#4	c-stage III cT3N2M0	Middle	DCF 2 cycles +CRT	No	pT4b(Br)N2M0	Grade 1a	Typical BLR case. Adhesion area of trachea is not as wide as T4 Finally not curative resection
#5	c-stage III cT3N2M0	Middle	DCF 3 cycles	No	pT4b(Ao)N2M0	Grade 1a	Typical BLR case (relatively T3). Adhesion area was relatively small to aorta

Imaging with esophagoscopy, CT, and esophagography was conducted. Information about patient characteristics and the clinical course was not revealed to the evaluators. Evaluators were selected from members of a weekly oncology board for upper gastrointestinal disease. Twenty-five doctors answered this evaluation study. Information about the evaluators is shown in Table 2. This study was approved by the institutional review board at the Keio University School of Medicine (20160048).

**Table 2:** Characteristics of evaluators

Evaluator	N	Career year (mean, median)
Surgeon: staff	6	17–27 (21.2, 20)
Surgeon: non-staff	14	5–11 (6.9, 6)
Medical oncologist	4	6–25 (17, 18.5)
Imaging radiologist	1	27
Overall	25	5–27 (12.7, 8)

### Evaluation

Each evaluator provided an opinion in an independent manner. The first question was the clinical diagnosis before chemotherapy. The evaluator could select from four answers: definite T4, suspected T4, suspected T3, and definite T3. The second question was about resectability after chemotherapy. The evaluator could select from three answers: resectable (curative), hard to decide, and unresectable.

### Assessment of inter-evaluator heterogeneity

IEH was assessed by agreement of T3 or T4. Definite T4 and suspected T4 were classified as clinical T4. Suspected T3 and definite T4 were classified as clinical T3. IEH was also compared for occupational differences. The clinical diagnosis of the staff surgeons, non-staff surgeons, and non-surgeon (medical oncologists and imaging radiologist) was compared. A discrepancy was defined as the clinical T4 converted to resectable.

## Results

### General IEH of clinical diagnosis

Overall results are summarized in Table 3. In case #1, IEH was observed for clinical diagnosis before chemotherapy (clinical T4: 52%, clinical T3: 48%). In cases #2–#5, most evaluators diagnosed it as clinical T4 with 76–88% agreement and no evaluators diagnosed it as definitive T3. IEH for clinical diagnosis of resectability was relatively small. For cases #1–#3, more than 70% of evaluators answered resectable; for cases #4 and #5, only 13 and 17% evaluators answered resectable, respectively. Answers of unknown were increased in cases #4 and #5 (54%). Comparing clinical diagnoses before and after chemotherapy, cases #2 and #3 were converted to resectable after the initial treatment, although the clinical diagnosis from evaluators was T4 before chemotherapy (*p* < 0.001). Cases #4 and #5 were not converted to resectable (*p* = 1.000 and 0.250, respectively).

**Table 3:** Summary of the study

Evaluation	Finally resected (%)			Finally not resected (%)	
	#1	#2	#3	#4	#5
Clinical diagnosis (before chemotherapy)					
Definite T4	20	56	44	32	36
Suspect T4	32	32	36	56	40
Clinical T4 (definite T4 + suspect T4)	52	88	80	88	76
Suspect T3	40	12	20	12	24
Definite T3	8	0	0	0	0
Clinical T3 (suspect T3 + definite T3)	48	12	20	12	24
Clinical diagnosis of resectability (after chemotherapy)					
Resectable (R0)	76	76	72	8	12
Hard to decide	20	12	20	28	28
Unresectable	4	12	8	64	60

### IEH by occupations

**Fig. 7:**
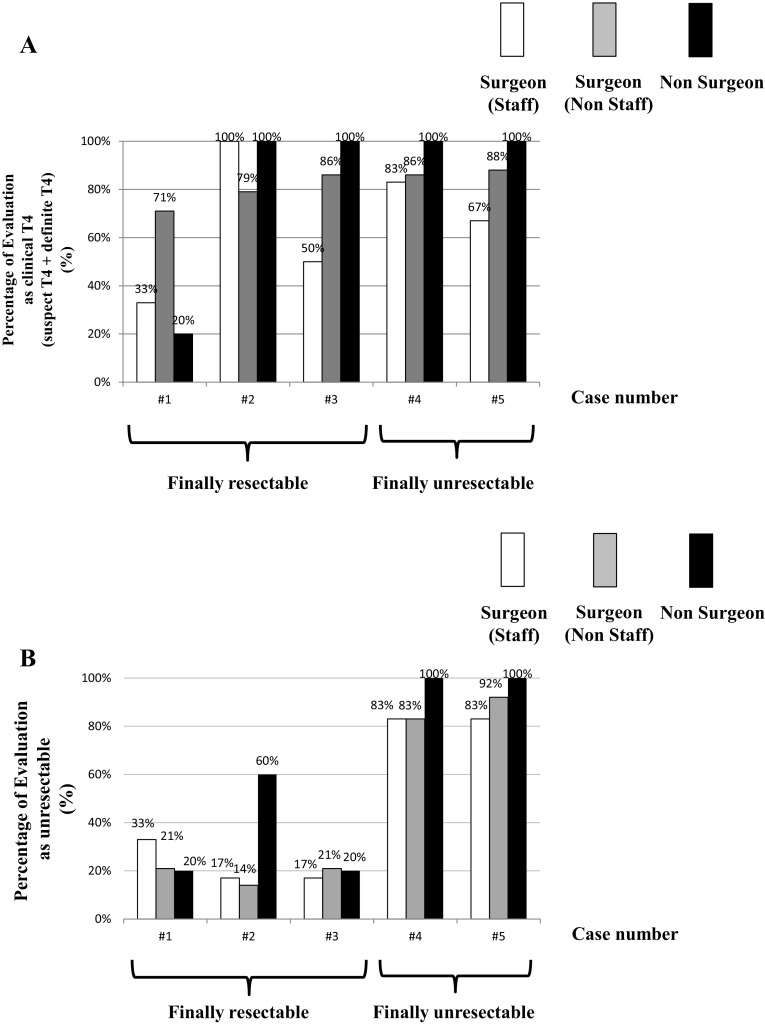
**a** Inter-evaluator heterogeneity of clinical T diagnosis. **b** Inter-evaluator heterogeneity of clinical judgment for resectability

Occupational differences are shown in Fig. 7. IEH before chemotherapy was also observed. In case #3, clinical evaluations of T stage were split (#3: 50%) among staff surgeons. Clinical evaluations were also split into cases #1 and #5; however, the difference was relatively small (#1: 33%, #5: 67%). IEH between occupations was observed in case #1. Most non-staff surgeons evaluated it as T4, although staff surgeons, medical oncologists, and the imaging radiologist did not evaluate it as T4. IEH for resectability after chemotherapy was observed in case #2 by medical oncologists. Other heterogeneity was relatively small compared to the evaluation of the imaging radiologist.

## Discussion

In our study, several types of IEH in locally advanced ESCC were observed. Occupational differences were seen even among experienced surgical oncologists. However, most of the differences in clinical judgments were relatively small. The depth of invasion of esophageal cancer needs to consider in the direction of the ulcer. The CT images need to adjust with information provided by endoscopic findings [16]. This method with combined evaluation of CT and endoscopy for T4 diagnosis is widely known in matured surgical and medical oncologist for esophageal cancer, however, not familiar in less experienced doctors.

IEH was observed in each occupation. First, non-staff surgeons over-estimated T3 disease (case #1). Case #1 was selected as a typical T3, and most staff surgeons, medical oncologists, and the imaging radiologist did not evaluate it as T4. This case was lower thorax cancer with aorta invasion suspected. However, adhesion area was not as wide as definitive T4. This IEH could explain the experimental heterogeneity of locally advanced ESCC. This kind of IEH could be expected in law-volume centers for ESCC [17–19]. Second, occupational differences were observed between medical oncologists and other occupations for clinical resectability after chemotherapy. In case #2, most surgeons, including non-staff, evaluated it as resectable, although most medical oncologists did not consider it resectable. This case was finally resectable, and therefore, judgment of resectability seems established in surgical oncologists. It was also explained that attitude of each professional could affect the judgments. This IEH could also explain the conservative attitude of medical oncologists for converting to surgery. These two IEH could be overcome by educational programs and multi-disciplinary team activity through an oncology board.

Finally, there was IEH among staff surgeons for cases #1 and #3. This IEH reflected the fact that the definition of T stage is not mature. Especially, difference of trachea or aorta invasion is important. In general, T4 diagnosis of trachea/bronchus is rather difficult. Half of the staff surgeon was matured esophageal surgical oncologists; however, half of them were upper gastrointestinal surgeon mainly for stomach. To overcome this IEH, education and more experience for esophageal cancer treatments are important solution. However, it may be insufficient. Categorization and clarification of BLR, if possible, is one of good resolution. Another consideration for solving this problem is including BLR cases to T3 or T4 disease in clinical practice. After completion of neo-adjuvant treatment, re-evaluate resectability with multi-disciplinary team. It could also evaluate with clinical trial. After completion of enrollment, pre-planned subgroup analysis with BLR and definitive T4 cases will find some answers. A well-designed clinical trial could have the potential to solve the problem.

Our study clearly shows that clinical diagnoses ‘before chemotherapy’ were not conclusive; however, IEH ‘after chemotherapy’ was relatively small and reliable. We have already reported a prospective trial of the triplet regimen for ESCC clinical T4 cases, including BLR [19]. This study investigated the efficacy and safety of an initial DCF regimen followed by selected surgery if possible. As far as we know, this phase 2 trial is the first clinical trial for ESCC to include BLR disease. We are now planning to conduct a phase 3 trial to evaluate neo-adjuvant DCF treatment for clinical T4 cases, including BLR cases, in JEOG.

Our study has several limitations to consider. First, this study is a single institution analysis and there were imbalances of profession. Non-surgical evaluator, especially imaging radiologist, was alone. We need to consider inter-professional bias in this study. Further confirmative study or other method is needed to clarify this bias. Second, the number of evaluated cases was small; only five cases were chosen from a single institution. In addition, we need to consider memory bias. Third, the case selection by investigator had a potential bias. Finally, it is possible that these questionnaires itself mislead the evaluators’ answers. It is also important educational aspect of questionnaires during question through case #1 to case #5.

In conclusion, IEH of evaluating BLR cases in ESCC needs to be considered. A multi-disciplinary team is essential to overcome this problem.
